# Continuous Separation of Lithium Iron Phosphate and Graphite Microparticles via Coupled Electric and Magnetic Fields

**DOI:** 10.3390/mi16101094

**Published:** 2025-09-26

**Authors:** Wenbo Liu, Xiaolei Chen, Pengfei Qi, Xiaomin Liu, Yan Wang

**Affiliations:** 1 College of Chemistry and Chemical Engineering, Qingdao University, Qingdao 266071, China; 13949827138@163.com (W.L.); ccchenxl1210@163.com (X.C.); 2 State Key Laboratory of Bio-Fiber and Eco-textiles, Collaborative Innovation Center for Marine Biobased Fibers and Ecological Textiles, Institute of Marine Biobased Materials, College of Materials Science and Engineering, Qingdao University, Qingdao 266071, China; pengfei@uni-bremen.de

**Keywords:** dielectrophoresis, magnetophoresis, numerical simulation, continuous separation, lithium iron phosphate and graphite

## Abstract

Driven by the growing demand for sustainable resource utilization, the recovery of valuable constituents from spent lithium-ion batteries (LIBs) has attracted considerable attention, whereas conventional recycling processes remain energy-intensive, inefficient, and environmentally detrimental. Herein, an efficient and environmentally benign separation strategy integrating dielectrophoresis (DEP) and magnetophoresis (MAP) is proposed for isolating the primary components of “black mass” from spent LIBs, i.e., lithium iron phosphate (LFP) and graphite microparticles. A coupled electric–magnetic–fluid dynamic model is established to predict particle motion behavior, and a custom-designed microparticle separator is developed for continuous LFP–graphite separation. Numerical simulations are performed to analyze microparticle trajectories under mutual effects of DEP and MAP and to evaluate the feasibility of binary separation. Structural optimization revealed that the optimal separator configuration comprised an electrode spacing of 2 mm and a ferromagnetic body length of 5 mm with 3 mm spacing. Additionally, a numerical study also found that an auxiliary flow velocity ratio of 3 resulted in the best particle focusing effect. Furthermore, the effects of key operational parameters, including electric and magnetic field strengths and flow velocity, on particle migration were systematically investigated. The findings revealed that these factors significantly enhanced the lateral migration disparity between LFP and graphite within the separation channel, thereby enabling complete separation of LFP particles with high purity and recovery under optimized conditions. Overall, this study provides a theoretical foundation for the development of high-performance and environmentally sustainable LIBs recovery technologies.

## 1. Introduction

With the global energy structure transitioning toward low-carbon alternatives, increasing emphasis is being placed worldwide on the development of green, environmentally friendly, and renewable energy sources [1,2]. Driven by continuous advancements and technological innovation, the new energy vehicle (NEV) market has expanded rapidly in recent years. As the core component of NEVs, energy storage systems, and portable electronic devices, lithium-ion batteries (LIBs) have experienced explosive growth within the industry [3,4]. From 2015 to 2040, the production of LIBs for electric vehicles is projected to increase from 0.33 million tons to 4 million tons [5]. Meanwhile, with the approaching peak in battery retirement [6,7,8], the recycling of millions of spent LIBs has become a global concern. In 2020, China alone generated approximately 313,300 tons of spent lithium iron phosphate (LFP) batteries, and this number continues to increase annually [9,10,11,12]. If not properly managed, these batteries pose serious threats to the ecological environment and may result in the loss of valuable strategic metals [13].

LFP, as one of the earliest cathode materials to be commercially adopted at scale in electric vehicles, has become a core cathode material for both electric vehicles and energy storage systems due to its excellent thermal stability, long cycle life, and low cost [14,15]. However, during long-term charge–discharge cycling, LFP cathode materials tend to develop lithium vacancy defects and iron–lithium antisite defects, which are the primary causes of the decline in cathode material activity in LFP batteries [16,17,18]. Fortunately, for LFP materials with relatively minor degradation, the particle morphology and crystal structure remain nearly unchanged, making direct regeneration a feasible approach. The typical recycling process for spent lithium-ion batteries can be divided into two major stages: pretreatment and post-treatment. The pretreatment stage primarily involves discharging, dismantling, crushing, and sorting, and this phase is relatively mature in terms of technological development. The post-treatment stage mainly includes pyrometallurgy, hydrometallurgy, direct regeneration, and bioleaching [19,20,21,22]. Dang et al. [23] added CaCl_2_ to smelting slag and conducted roasting at 800 °C for 1 h, converting insoluble lithium into water-soluble salts. Lithium was then recovered through a water leaching process, achieving a leaching rate as high as 90%. In addition to CaCl_2_, Li et al. [24] demonstrated that the addition of Na_2_SO_4_ could also accomplish this transformation. Although pyrometallurgical techniques have undergone continuous optimization, issues such as high energy consumption, elevated reaction temperatures, and significant emissions remain major challenges. Yang et al. [25] successfully synthesized a NiCoMn layered double-hydroxide (LDH) catalyst via a co-precipitation method using leachate from spent lithium battery cathode materials, significantly reducing the cost of the recycling process. However, most inorganic acids used in metal leaching tend to generate toxic gases such as Cl_2_, SO_3_, and NO during the process, leading to secondary pollution. Fan et al. [26] employed a natural organic acid, i.e., malonic acid, in combination with H_2_O_2_ to leach spent LiNixMnyCozO2 (NCMs) materials. Srinivasan et al. [13] utilized bioleaching techniques with autotrophic bacteria such as *A. ferrooxidans* to recover valuable metals from sulfide sources. Nevertheless, the use of acids inevitably raises concerns over wastewater treatment, which contributes to environmental pollution and higher operational costs. Therefore, for LFP materials with severely degraded electrochemical performance, it is imperative to develop simpler and more efficient recycling methods that are both environmentally friendly and sustainable.

Dielectrophoresis (DEP) separation is a technique based on the differential dielectrophoretic forces experienced by particles in a non-uniform electric field. This method is characterized by high precision, strong flexibility, and label-free operation [27,28,29,30,31]. Yang et al. [32] applied voltage pulses to generate localized non-uniform electric fields, enabling the reconnection of isolated lithium–silicon (LixSi) particles back into the conductive network while simultaneously recovering the lost active material from Si electrodes. In our previous work [14], we developed a DEP-based separator for the continuous isolation of LFP from graphite. Both simulation and experimental results confirmed that low voltage facilitated the favorable separation of LFP particles, achieving a separation efficiency exceeding 80% at 100 V. At higher voltages, however, enhanced lateral displacements of graphite particles promoted their separation. Further analysis revealed that excessively high input voltages induced strong repulsive forces on some LFP particles as they entered the effective separation zone, forcing them toward the channel wall and preventing re-entry into the separation region, ultimately resulting in a marked reduction in selectivity. On the other hand, magnetophoresis (MAP) separation technique employs magnetic fields to separate magnetic particles or magnetically labeled materials, offering high selectivity and recovery efficiency [33,34,35]. In a recent study, Hu et al. [36] employed a high-intensity magnetic field to separate LFP and graphite, achieving a recovery rate of up to 96.6%. Nevertheless, this approach was limited in that only LFP particles within the high-gradient magnetic field region could be captured, while unintended inclusion of non-magnetic particles compromised the capture efficiency of LFP. Inspired by these studies, we hypothesize that integrating DEP and MAP could overcome the inherent limitations of either technique alone, thereby exploiting their synergistic effects to significantly improve the separation performance of spent electrode materials. To the best of our knowledge, however, reports on the combined application of DEP and MAP for the continuous separation of LFP and graphite remain scarce.

The main objective of this study is to investigate the feasibility and design strategies of using DEP-MAP technology for the recovery of spent battery materials. A microfluidic DEP-MAP separator capable of continuously separating LFP from graphite was designed. Coupled fluid flow, electric field, and magnetic field models were established, and the Lagrangian particle tracking method was employed to predict particle trajectories within the separator. The effects of separator structural and operating parameters were numerically examined to enhance separation accuracy and efficiency.

## 2. Theory and Numerical Simulations

### 2.1. Separation Mechanism

**Figure 1:**
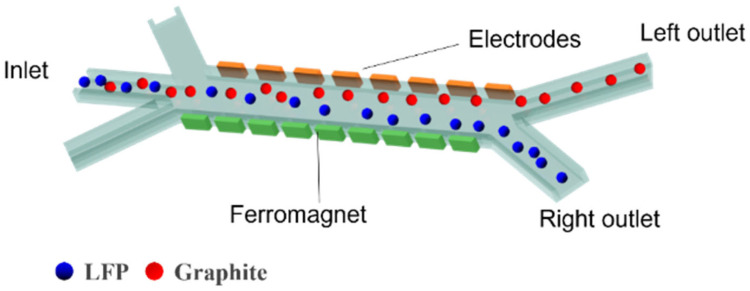
Schematic diagram of the separator design.

**Figure 2:**
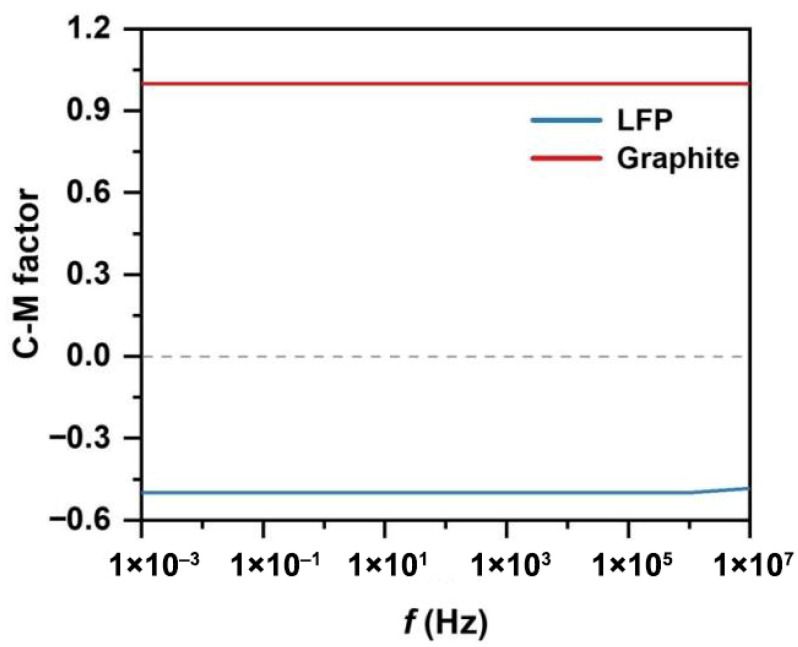
Clausius–Mossotti factor for separating particles in water (conductivity of 0.055 µS/cm).

The separation channel consists of a sample inlet region, a MAP and DEP sorting region, and an outlet collection region. Under the influence of sheath flows on both sides of the sample inlet region, the mixed particles are directed into the MAP and DEP sorting zone. In this region, due to the combined effects of DEP and MAP, LFP and graphite particles exhibit differential motion in distinct directions. Figure 1 illustrates the separation of LFP particles from graphite using a combined MAP and DEP approach within the separation channel. The mixed sample enters the central separation channel, where differences in magnetic susceptibility and electrical conductivity between the two types of particles are exploited. As shown in Figure 2, due to the distinct dielectric property differences between LFP and graphite particles, the two experience DEP forces of opposite directions and varying magnitudes over a wide frequency range. LFP particles exhibit negative DEP forces along with attractive MAP forces induced by the non-uniform magnetic field, resulting in a vector combination of the two forces that enables effective separation. When exposed to a non-uniform electric field, such strong polarization typically causes graphite particles to exhibit positive DEP forces [37]. Therefore, the separation channel is designed such that the two types of particles are directed toward different outlets on either side, enabling effective separation.

When a non-uniform electric field is applied to the system, the average dielectrophoretic force acting on a spherical particle (radius *r*) can be approximated as [38,39,40]:

(1) $$F_{DEP}=2\pi \varepsilon_{m}r^{3}Re[f_{CM}(\omega )]\nabla |E_{rms}{|}^{2}$$

where *r* is the radius of the spherical particle, $\varepsilon_{m}$ is the dielectric constant of the suspending medium, $\nabla |E_{rms}{|}^{2}$ is the square of the electric field gradient, and $Re[f_{CM}(\omega )]$ is the real part of the Clausius–Mossotti (CM) factor, which describes the relative polarization of the particle and the suspending medium. This factor is a function of both the particle’s geometry and the frequency [41,42].

(2) $$f_{CM}(\omega )=\frac{\varepsilon_{p}^{*}-\varepsilon_{m}^{*}}{\varepsilon_{p}^{*}+2\varepsilon_{m}^{*}}$$

where $\varepsilon_{p}^{*}$ and $\varepsilon_{m}^{*}$ denote the complex permittivity of the particle and the suspending medium, respectively. The complex permittivity is defined as $\varepsilon^{*}=\varepsilon -(j\sigma /\omega )$, where ε is the dielectric constant of the LFP or the solution, σ is the corresponding electrical conductivity, $j=\sqrt{-1}$ is the imaginary unit, and ω = 2πf is the angular frequency of the applied electric field, with f being the conventional (linear) frequency.

As indicated by Equations (1) and (2), the magnitude of the DEP is related to the particle size $r^{3}$ and the gradient of the non-uniform electric field $\nabla |E_{rms}{|}^{2}$, while the direction of the particle’s DEP trajectory is determined by the Clausius–Mossotti factor $f_{CM}(\omega )$. If the polarizability of the LFP is greater than that of the surrounding medium, the Clausius–Mossotti (CM) factor is positive, and the particle experiences a positive dielectrophoretic (p-DEP) force, which attracts it toward regions of higher electric field intensity. Conversely, when the CM factor is negative, the particle is subjected to a negative dielectrophoretic (n-DEP) force and is repelled toward regions of lower electric field intensity. Due to variations in size and intrinsic properties, different particles follow distinct trajectories and are directed toward different outlets within the separation channel.

MAP refers to the directional movement of particles under the influence of a magnetic field. The MAP force acting on a spherical particle with radius *r* can be defined as [43]:

(3) $$F_{MAP}=\frac{4}{3}u_{m}u_{0}\pi r^{3}K(H\cdot \nabla )H$$

where $u_{m}$ is the relative magnetic permeability of the suspending medium, $u_{0}=4\pi \times 10^{-7}$, is the permeability of free space (vacuum), and ***H*** is the strength of the applied magnetic field. The parameter *K* is defined as follows:

(4) $$K=3(\chi_{P}-\chi_{m})/[(\chi_{P}-\chi_{m})+3(\chi_{m}+1)]$$

where $\chi_{P}$ denotes the magnetic susceptibility of the particle, and $\chi_{p}=\mu_{p}-1$, where $\mu_{p}$ is the relative permeability of the particle. $\chi_{m}$ represents the magnetic susceptibility of the suspending medium. When $\chi_{p}>\chi_{m}$, the particle is subjected to a positive magnetophoretic (p-MAP) force and is drawn toward the high-field-strength region of the non-uniform magnetic field. Conversely, when the magnetic susceptibility of the particle is lower than that of the surrounding suspending medium, a negative magnetophoretic (n-MAP) force acts on the particle, pulling it toward the low-field-strength region of the non-uniform magnetic field.

### 2.2. Physical and Mathematical Models

**Figure 3:**
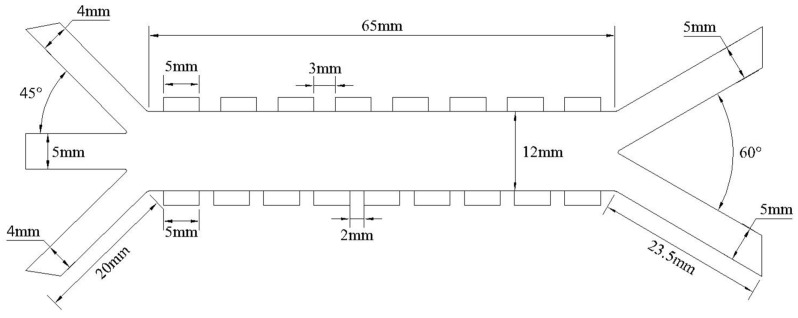
CAD drawing of the separator.

The microchannel inlet consists of sheath flow inlets on both sides and a central inlet for the mixed particle suspension. To focus the particles in a region favorable for separation, the angle between the sheath flows (inlets 1 and 3 in Figure 3) and the sample inlet (inlet 2 in Figure 3) is set to 45°. Driven by the sheath flow, the sample stream enters the separation channel. Under the combined effects of DEP and MAP, particles undergo lateral displacement. LFP particles, subjected to nDEP, are deflected toward and exit from the right outlet, while graphite particles, influenced by pDEP, migrate toward the left outlet. The detailed structural dimensions of the separator are shown in Figure 3.

#### 2.2.1. Hydrodynamics

The fluid flow within the separation channel is considered incompressible and laminar. Based on the calculated Reynolds number, the inertial forces are significantly smaller than the viscous forces and thus can be neglected. A relatively low frequency (f = 1 kHz) was used, and the suspending medium was ultrapure water with extremely low conductivity; under these conditions, the thermoelectric forces generated by Joule heating can be neglected. The flow is governed by the Navier–Stokes equations [44]:

(5) $$\rho u\cdot \nabla u=-\nabla P+\mu \nabla^{2}u+\rho g$$

The continuity equation is given by:

(6) ρ∇u=0

where u is the velocity vector, and ρ and μ are the density and viscosity of the medium, respectively. ∇P represents the pressure gradient within the flow field. Additionally, under steady-state conditions, the flow field does not change with time, and the channel walls are assumed to be slip boundaries.

#### 2.2.2. Magnetic Field

The magnetic field within the separation channel is governed by Maxwell’s equations [45].

(7) ∇×H=J

(8) $$H=-\nabla V_{m}+H_{b}$$

where H denotes the magnetic field strength, $V_{m}$ is the magnetic scalar potential, and $H_{b}$ represents the external magnetic field strength. According to Gauss’s law for magnetism, the magnetic flux density B is expressed as:

(9) ∇⋅B=0

#### 2.2.3. Electric Field

The distribution of the applied external electric field is governed by Laplace’s equation [46]:

(10) $$\nabla^{2}\phi =0$$

where $\phi_{max}=U_{max}$ represents the electric potential, with $U_{max}$ being the peak voltage applied to the electrodes. Opposing voltages are applied to the external electrodes.

#### 2.2.4. Particle Tracking

To describe the trajectories of various particles within the separator, particle motion is coupled with the electric field, magnetic field, and fluid dynamics. The particle tracking is governed by Newton’s second law:

(11) $$m_{p}\frac{dv}{dt}=F_{t}$$

where $m_{p}$ and v are the mass and velocity of the particle, respectively. $F_{t}$ is the total force acting on the particle, including drag force, gravitational force, MAP, and DEP. At sufficiently low particle concentrations, the interparticle interaction forces can be reasonably neglected.

Due to the viscosity of the fluid, particles experience drag when moving through the sheath flow. Under laminar flow conditions, the drag force acting on each particle is described by Stokes’ drag law:

(12) $$F_{d}=\frac{1}{\tau_{p}}m_{p}\left(u-v\right)$$

(13) $$\tau_{p}=\frac{\rho_{p}d_{p}^{2}}{18\mu }$$

where $\rho_{p}$ is the density of the particle, and $d_{p}=2r$ is the diameter of the spherical particle.

In this study, both types of particles have densities greater than that of the fluid medium; therefore, the gravitational force is given by:

(14) $$F_{g}=m_{p}g\frac{\rho_{p}-\rho }{\rho_{p}}$$

The DEP and MAP forces acting on the particles are given by Equations (1) and (3), respectively.

To quantify the separation performance of the designed separator, the separation efficiency ($\eta_{LFP}$) and purity ($\gamma_{LFP}$) of the LFP from mixed particles were calculated using the mass (*m*_i,j_) and number (*n*_i,j_) of i particles at the j outlet, and the recovery (*Rec*) of all particles is defined as the ratio of the number of particles of i particles ($Z_{i}^{\prime }$) reaching the target outlet channel to the total number of i particles $Z_{i}$ collected at both outlet channels, as follows:

(15) $$\eta_{LFP}=\frac{m_{LEP,2}}{m_{LEP,1}+m_{LEP,2}}\times 100\%$$

(16) $$\gamma_{LFP}=\frac{n_{LEP,2}}{n_{LEP,2}+n_{c,2}}\times 100\%$$

(17) $${Rec}_{LFP}=\frac{\sum Z_{i}^{\prime }}{\sum Z_{i}}\times 100\%$$

### 2.3. Computational Domain and Boundary Conditions

The model employs a particle tracing module based on the Lagrangian approach, coupled with the effects of electric, magnetic, and fluid fields on the total volumetric forces acting on the particles. In the particle-tracing module, the central inlet is defined as the inlet boundary for the mixed particles, while the left and right outlets serve as the outlet boundaries for the two types of particles. The glass channel walls and electrode surfaces within the domain are set as “bounce” boundaries. Table 1 summarizes the computational domain and boundary conditions defined in COMSOL Multiphysics (v6.2).

### 2.4. Numerical Simulation

In this study, COMSOL Multiphysics^®^ 6.2 was used to solve the above equations and boundary conditions for the numerical simulation of LFP and graphite particle separation [36,47]. First, a stationary solver was used to compute the flow field, electric field, and magnetic field. Then, particle tracking was employed to introduce the particle mixture into the separator, and the trajectories of the mixed particles were calculated under the combined effects of MAP and DEP forces. The effects of electrode and ferromagnetic structure positions, the flow rate ratio between the sheath flows and particle flow, magnetic field strength, and applied voltage on particle trajectories were also investigated. The parameter values used in the simulations are listed in Table 2 [48].

To investigate the separation efficiency, the parameter values of the particles used in the separation process are listed in Table 3.

### 2.5. Grid Independence Verification

**Figure 4:**
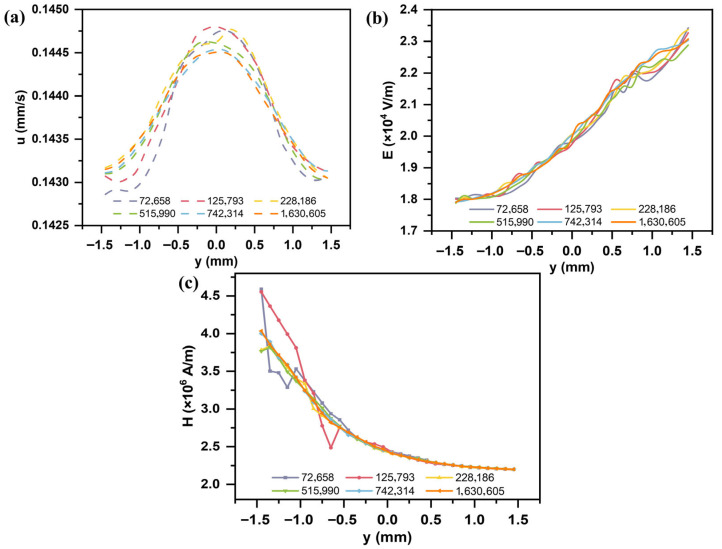
Grid independence verification of (**a**) velocity, (**b**) electric field, and (**c**) magnetic field.

A grid independence study was conducted for the flow field, electric field, and magnetic field to ensure reliable simulation results that are independent of mesh size (Figure 4). Simulations were performed using different numbers of mesh elements: 72,658; 125,793; 228,186; 515,990; 742,314; and 1,630,605. When the mesh size reached 742,314 elements, the maximum relative deviations in the flow field, electric field, and magnetic field distributions were 0.05%, 1.1%, and 1.29%, respectively. Further increasing the mesh density resulted in negligible improvements in accuracy but caused an exponential increase in computational cost. Considering both computational efficiency and accuracy, all subsequent simulations in this study were performed using a mesh with 742,314 elements.

## 3. Results and Discussion

**Figure 5:**
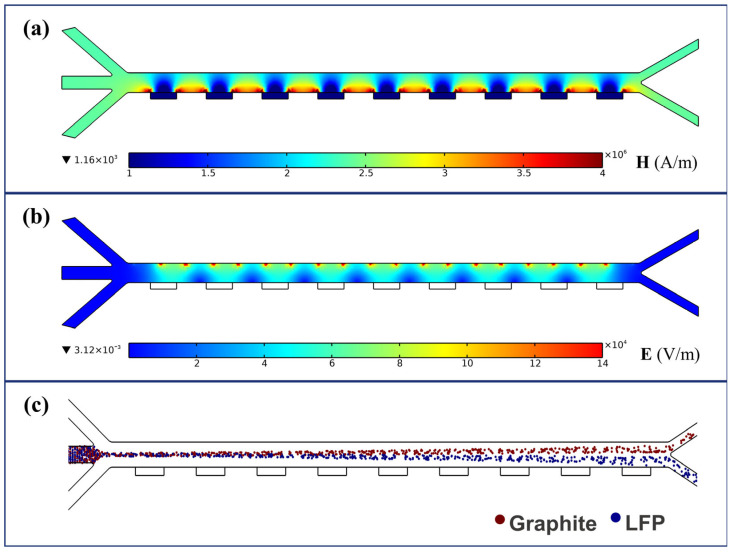
Numerical simulation of field distributions: (**a**) magnetic field; (**b**) electric field; (**c**) particle trajectories (with ***B*** = 2.4 T, $U_{eff}$ = 200 V, ***u*** = 30 mm/s, and *f* = 1 kHz).

Based on the proposed separator model, numerical simulations were conducted under an arrayed configuration of external field components. The magnetic field distribution is shown in Figure 5a, where higher magnetic field intensity appears between the ferromagnetic blocks located on the side of the channel opposite the electrodes. These regions exhibit a significantly enhanced magnetic field strength, which further increases the lateral driving force acting on LFP particles. The electric field distribution, shown in Figure 5b, presents a stepwise non-uniform pattern that covers the majority of the channel area. Regions of high electric field strength are concentrated near the edges of the electrodes between the positive and negative poles, while the electric field intensity decreases with increasing distance from the electrode surfaces. The combined distributions of the magnetic and electric fields ensure the effectiveness of the applied external fields throughout the entire channel, maximizing the influence on particle trajectory alteration. Due to the use of slip boundary conditions at the channel walls, the fluid exhibits a uniform distribution within the separation channel. Under the combined effects of the flow field and external fields, the particle trajectories shown in Figure 5c were obtained. It can be observed that hydrodynamic focusing significantly influences the movement paths of the particles.

### 3.1. Influence of Electrode and Magnet Configuration

#### 3.1.1. Effect of Electrode Spacing

**Figure 6:**
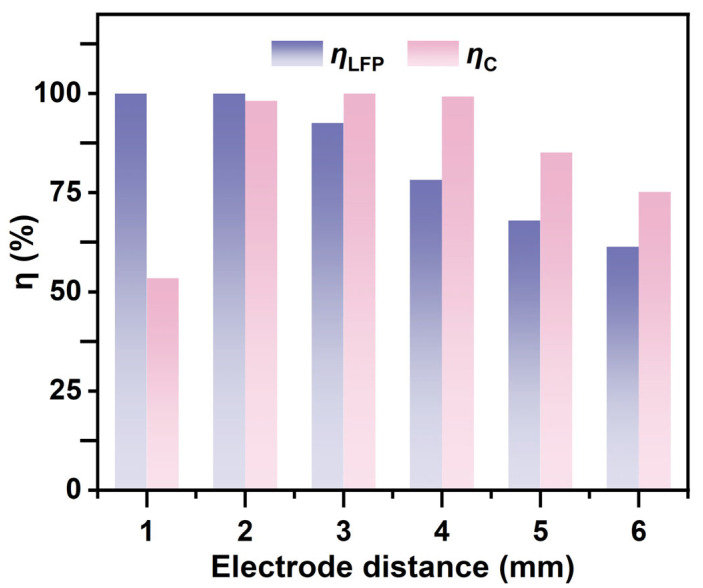
Separation efficiencies of LFP and graphite under different electrode spacings (with ***B*** = 2.4 T, $U_{eff}$ = 200 V, ***u*** = 30 mm/s, and *f* = 1 kHz).

The influence of electrode spacing on the DEP force is primarily reflected in the variations in electric field strength and its gradient. As shown in Figure 6, the influence of electrode spacing on the DEP force is primarily reflected in changes to the electric field strength and its gradient. As shown in Figure 6, the separation efficiency of LFP increases with increasing electrode spacing. However, excessively large electrode spacing significantly reduces the separation efficiency of graphite particles. This is because, under a constant applied voltage, a larger spacing leads to a pronounced attenuation of the electric field strength, resulting in insufficient effective DEP forces acting on the particles, thereby hindering efficient separation. In contrast, although smaller electrode spacing yields the highest electric field strength, it may intensify fluid disturbances and enhance particle–particle interactions, which interfere with the selectivity of separation. More importantly, excessively strong local electric fields increase the non-uniformity of the field, making edge effects more pronounced. Therefore, an electrode spacing of 2 mm was selected for further investigation.

#### 3.1.2. Effect of Ferromagnet Length

**Figure 7:**
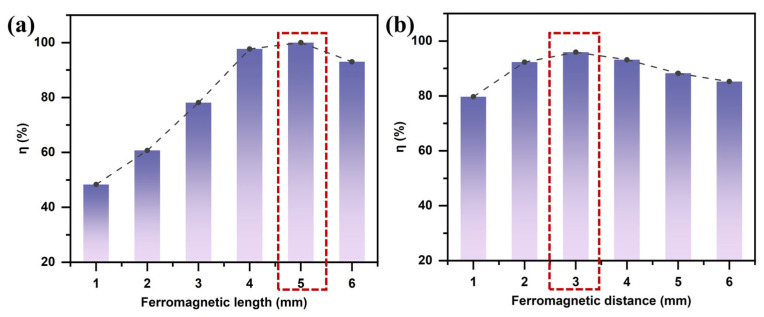
Effect of ferromagnetic structure on the separation efficiency of lithium iron phosphate (LFP): (**a**) ferromagnet length; (**b**) ferromagnet spacing (with ***B*** = 2.4 T, $U_{eff}$ = 200 V, ***u*** = 30 mm/s, and *f* = 1 kHz).

To investigate the influence of the ferromagnetic structure on the magnetic field gradient, numerical simulations were conducted by varying the length of the ferromagnetic blocks. As shown in Figure 7a, the length of the ferromagnetic component affects the separation efficiency of LFP primarily by regulating the magnetic field gradient. According to Equation (3), the magnitude of the MAP force is directly proportional to the square of the magnetic field gradient. The length of the ferromagnetic block alters the spatial characteristics of the magnetic field distribution. Near the magnetic poles, the magnetic field exhibits rapid spatial variation. When the ferromagnetic block is short, the poles are closely spaced, generating a strong MAP force in the vicinity of the poles. However, due to the rapid decay of the magnetic field intensity with distance, the effective range of the magnetic force is limited, making it ineffective for particles farther from the poles. As the length of the ferromagnetic block decreases below 5 mm, the separation efficiency of LFP gradually declines. Conversely, increasing the length of the block leads to a smoother magnetic field distribution with a reduced magnetic field gradient, thereby weakening the MAP force and reducing separation efficiency. Therefore, a ferromagnetic block length of 5 mm was selected to achieve optimal separation performance.

#### 3.1.3. Effect of Ferromagnet Spacing

The effect of ferromagnet spacing on particle separation is essentially realized through changes in the spatial distribution of the magnetic field gradient. When multiple ferromagnetic blocks are present, increasing the spacing results in a smoother magnetic field distribution. In contrast, smaller spacing leads to increased overlap between adjacent magnetic fields, generating localized regions of high magnetic field gradients near the magnetic poles and enhancing the MAP force. However, this enhancement comes at the cost of a significantly reduced effective magnetic field range. As shown in Figure 7b, the high-gradient regions are primarily confined to the sides of individual ferromagnetic blocks and are mostly located between adjacent magnets. Therefore, excessively small ferromagnet spacing restricts the effective separation zone and may also cause local particle trapping due to the high magnetic field strength, which ultimately reduces the overall separation efficiency for LFP particles.

### 3.2. Sheath-to-Sample Flow Rate Ratio

**Figure 8:**
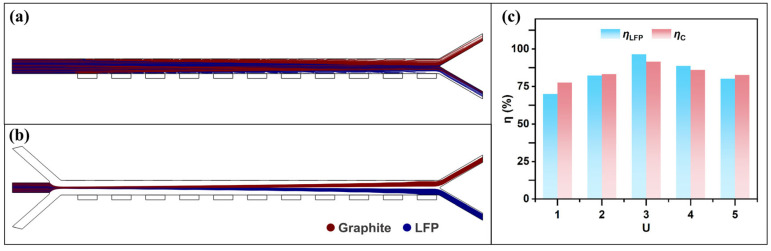
Effect of sheath flow on the particle separation process: (**a**) particle trajectories without sheath flow; (**b**) particle trajectories under dual-side sheath flow; (**c**) separation efficiencies of LFP and graphite at different flow rate ratios U (with ***B*** = 2.4 T, $U_{eff}$ = 200 V, ***u*** = 30 mm/s, and *f* = 1 kHz).

The sheath-to-sample flow rate ratio refers to the ratio of the particle flow rate to the flow rates of the sheath streams on both sides. Figure 8a,b show the particle trajectories with and without sheath flow, respectively. Particles located near the channel walls are easily captured under the influence of DEP and MAP forces. However, graphite particles on the side away from the electrodes and LFP particles on the side away from the ferromagnetic components experience weaker forces and thus fail to reach the desired outlet within the limited channel length. In this case, introducing sheath flows on both sides enables hydrodynamic focusing of the mixed particles, which effectively resolves the issue. To determine an appropriate sheath-to-sample flow rate ratio, the particle stream velocity was kept constant, while the base velocity of the sheath flow was varied to observe the changes in particle trajectories. Defining the sheath-to-sample flow rate ratio as U, Figure 8c shows the separation efficiency of LFP and graphite as a function of U in the range of 1 to 5. When U = 3, the separation efficiency of LFP and graphite reaches its maximum. This is because a lower flow rate ratio leads to a more dispersed distribution of the mixed particles as they enter the separation channel. Particles of the same type located at different lateral positions experience varying forces; as a result, some near the channel walls may already be captured by the electrodes, while others may not be sufficiently affected by the external forces. On the other hand, when the flow rate ratio is too high, the focused particle stream remains confined to the center of the channel. Within the limited channel length, many particles do not experience sufficient lateral deflection and exit through the undesired outlet.

### 3.3. Effect of Applied Magnetic Field Strength

**Figure 9:**
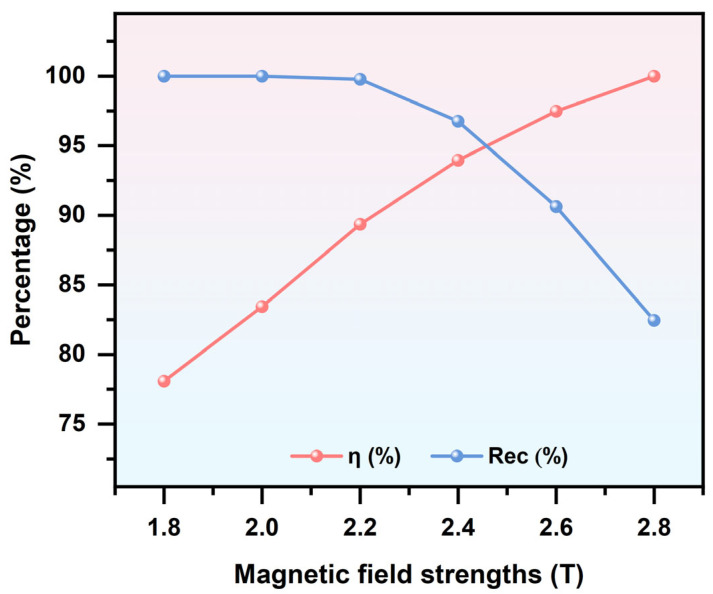
Separation efficiency and recovery rate of LFP under different applied magnetic field strengths. (with $U_{eff}$ = 100 V, ***u*** = 30 mm/s, and *f* = 1 kHz).

By applying an external magnetic flux density to magnetize the ferromagnetic blocks in the separation device, the MAP force acting on particles within the channel can be enhanced. Increasing the applied magnetic flux density further strengthens this effect. As shown in Figure 9, a decrease in the applied magnetic flux density results in a weaker MAP force, causing particles to follow trajectories primarily governed by DEP effects, thereby reducing the separation efficiency. In contrast, as the magnetic flux density increases, the degree of magnetization of the ferromagnetic blocks is enhanced, leading to stronger magnetic fields within the channel. Consequently, the MAP force acting on LFP particles becomes more pronounced, resulting in improved separation efficiency. However, when the applied magnetic flux density exceeds 2.2 T, the excessively strong magnetic field causes LFP particles to be attracted toward the vicinity of the ferromagnetic blocks, leading to increased particle adhesion and a reduction in recovery rate. Meanwhile, it is also important to consider the trade-off between external field strength and system energy consumption, as an overly high field intensity can significantly increase the system’s energy demand. Based on this, a magnetic flux density of ***B*** = 2.4 T was selected to ensure the effectiveness and accuracy of particle separation.

### 3.4. Relationship Between Voltage and Flow Rate

**Figure 10:**
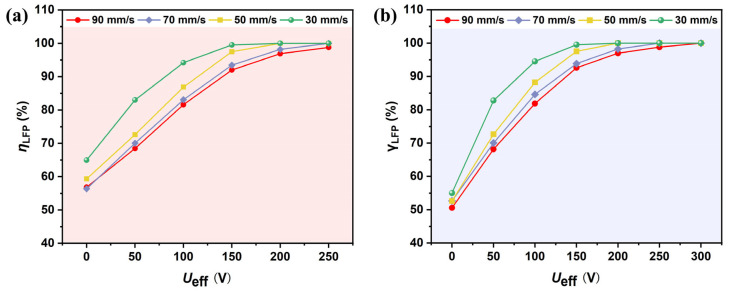
Effect of voltage and flow rate on the (**a**) separation efficiency and (**b**) purity of LFP (with ***B*** = 2.4 T, and *f* = 1 kHz).

To evaluate the effect of applied voltage and flow rate on the separation of graphite and LFP particles, simulations were conducted with various inlet velocities and applied voltages ranging from 100 V to 300 V. According to Equation (1), the DEP force is proportional to the gradient of the non-uniform electric field, which increases exponentially with higher applied voltage. Theoretically, the separation efficiency of particles is expected to improve with increasing voltage. As shown in Figure 10a, the particle separation efficiency is positively correlated with the applied voltage. When the voltage increases from 100 V to 300 V, the separation efficiency rises from 56.84% to 98.76%. This trend indicates that higher voltages significantly enhance the lateral dielectrophoretic migration of particles, thereby improving the separation performance. However, when the voltage exceeds 200 V, the efficiency growth tends to saturate, while the Joule heating effect intensifies. If the temperature rise exceeds 10 K, additional cooling systems may be required, leading to increased equipment and operational costs.

The analysis of flow rate effects reveals that particle velocity is positively correlated with the external force required to achieve effective separation. When the flow rate increases from 30 mm/s to 90 mm/s, the applied voltage must be raised by approximately 50% to maintain a comparable separation efficiency. This occurs because, at higher flow rates, the residence time of particles within the channel is shortened, thereby limiting the opportunity for sufficient lateral migration. Moreover, lower flow rates reduce the pumping power demand and significantly decrease the voltage threshold required for efficient separation, which is advantageous from an energy consumption perspective. To balance separation efficiency and energy economy, $U_{eff}$ = 200 V, ***u*** = 30 mm/s were ultimately selected as the optimal operating conditions. Under these conditions, the theoretical separation efficiency approaches 100%, achieving efficient separation while controlling energy costs and thus reaching a favorable balance in overall performance.

## 4. Conclusions

In this study, a numerical simulation of the separation of LFP and graphite particles was conducted using a coupled DEP-MAP method in a pressure-driven microfluidic flow. To enhance the magnetophoretic effect, multiple pairs of ferromagnetic segments were employed to generate high-gradient magnetic fields. The size and spacing of the ferromagnetic blocks were optimized to provide sufficient magnetic force. Structurally, a microfluidic separator with a three-inlet and two-outlet configuration was designed, in which sheath flows from both sides focused the mixed particles toward the center of the channel, significantly improving separation precision. Numerical results demonstrated that optimal electric and magnetic field gradients were achieved when the electrode spacing was 2 mm, ferromagnet length was 5 mm, and spacing was 3 mm, where the processing capacity of the separator was calculated to be 54 mL/min. In addition, appropriate combinations of voltage and flow rate were found to be critical for effective separation. The results indicate that, under optimized operating conditions (***B*** = 2.4 T, $U_{eff}$ = 200 V, ***u*** = 30 mm/s, *f* = 1 kHz), LFP particles can be efficiently separated, with the theoretical separation efficiency approaching 100% while maintaining high purity and high recovery. It should be noted that this result represents an ideal value obtained under the present simulation conditions, and its practical feasibility still requires further experimental validation. This work not only elucidates the effects of key parameters on separation performance but also demonstrates the feasibility of the proposed DEP–MAP coupling method for the separation of LFP and graphite particles. Moreover, the design concept and methodology can be extended to other particle separation systems with differences in electromagnetic properties, highlighting its broad application potential.

## Figures and Tables

**Table 1:** Computational Domains and Boundary Conditions Used in COMSOL Multiphysics.

	Computation Domain/Boundary Conditions (Scope of Application)	Governing Conditions/Equations
Laminar flow module	Entrance (Top to bottom)	$u_{s}$, u, $u_{s}$
	Export (Top to bottom)	P=0
	Slip (walls, electrodes)	u⋅n=0
Current module	Conservation of current	$\nabla \cdot J\;=Q_{J,\;V}J\;=\sigma E\;+J_{e}\;E\;=-\text{∇}\varphi$
	Electrical insulation (walls)	n⋅J=0
	Initial values (domain 1)	φ=0
	Potential 1 (electrodes 1 and 2)	$U_{0}$
	Potential 2 (electrodes 3 and 4)	−$U_{0}$
Magnetic module	Conservation of Magnetic	∇×H=J $H=-\nabla V_{m}+H_{b}$
	Initial values (domain 1)	∇⋅B=0
Particle tracking	Bounce (walls, electrodes)	$v=v_{c}\;-\;2(n\cdot v_{c})n$
	Entrance (entrance 2)	$q\;={\;q}_{0}$ $v=\;v_{0}$
	Freeze (exports 1, 2, and 3)	$v=v_{c}$

**Table 2:** Parameter values used in the simulation.

Parameters	Values
Dielectric constant of buffer liquid phase, $\varepsilon_{m}$	80
Permittivity of vacuum, $\varepsilon_{0}(F\cdot m^{-1})$	8.85 × 10^−12^
Vacuum permeability, $u_{0}(N\cdot A^{-2})$	4π × 10^−7^
Density of buffer, $\rho_{b}(kg\cdot m^{-3})$	1000
Dynamic viscosity of buffer, $\mu_{b}(Pa\cdot s)$	1 × 10^−3^
Electrical conductivity of buffer $\left(S\cdot m^{-1}\right)$	5.5 × 10^−6^
Magnetic susceptibility of buffer, $\chi_{m}(m^{3}\cdot {kg}^{-1})$	−9.05 × 10^−9^
Microchannel height (mm)	6

**Table 3:** Material properties used in the simulation [49].

	Relative Permittivity	$\mathrm{Electrical}\;\mathrm{Conductivity}\;(S\cdot m^{-1})$	Diameter (μm)	Density $\left(kg\cdot m^{-3}\right)$	Magnetic Susceptibility
LFP	10	1.67 × 10^−8^	75	1523	7.41 × 10^−7^
graphite	12.35	2 × 10^5^	75	2250	−8.4 × 10^−8^
water	80	5.5 × 10^−6^	-	1000	-

## Data Availability

Data will be made available upon request.
